# To What Extent Can Digitally-Mediated Team Communication in Children’s Physical Health and Mental Health Services Bring about Improved Outcomes? A Systematic Review

**DOI:** 10.1007/s10578-021-01183-w

**Published:** 2021-05-08

**Authors:** Lauren Stephanie Jones, Ailsa Russell, Emma Collis, Mark Brosnan

**Affiliations:** Centre for Applied Autism Research, Department of Psychology, University of Bath, Bath, BA2 7AY UK

**Keywords:** Digital communication, Clinical team, Children and young people, Health service delivery, Systematic review

## Abstract

Digital communication technologies can be used for team consultation, case management, and information sharing in health and mental health services for children and young people (CYP). The objective of the systematic review was to investigate the evidence as to whether digitally-mediated team communication for CYP improves outcomes. We searched PsycINFO, PubMed, Web of Science, and Cochrane Library for relevant studies. Results were synthesised narratively. Seven studies were identified from 439 initial records. Analysis highlighted that digitally-mediated team communication is generally valued by professionals for supporting practice and that there is overall satisfaction with the process. There was preliminary evidence (from one study) that clinical outcomes from digitally-mediated team communication are comparable to those achieved by a collaborative service model with direct specialist care to service users via digital communication technology. There is a need for further high-quality research into clinical outcomes and service user experience, as well as financial implications.

**Supplementary Information:**

The online version contains supplementary material available at 10.1007/s10578-021-01183-w.

## Introduction

Effective communication between professionals is a core process for enhancing coordination and clinical decision-making in health and mental health services [1]. A clinical team can be defined as a group of two or more professionals who interact regularly to exchange messages and work together towards a common goal (e.g., [1, 2]). Team communication ranges from information sharing and discussion of treatment strategies (i.e., consultation) to joint meetings which facilitate multidisciplinary case conceptualization and intervention planning (i.e., case management) [3, 4]. Digital communication technologies (e.g., telephone, email, and videoconference technology like Skype) can be used to facilitate team communication in today’s health and mental health services, with the aim to improve access to specialist guidance and increase the number and range of professionals that are able to meet in this modality [3–5]. In the current review, we use the term digitally-mediated team communication to describe a model of care in which specialists provide expertise to frontline workers regarding individual children and young people (CYP) via digital communication technologies (i.e., a consultation service model or low intensity service intervention) [3, 4]. Examples include telephonic professional-to-professional consultation and multidisciplinary case management via videoconferencing technology. This is especially relevant for professionals who are geographically distanced in rural locations, although it is now of widespread importance for professionals who are social distancing as part of the global response to COVID-19.

Team communication is recommended for addressing service user needs of varying severity and complexity [1, 4, 6]. This includes childhood health, mental health, behavioral, and developmental concerns. Communication between professionals from a range of disciplines (i.e., a multidisciplinary clinical team) is essential for a ‘whole-system’, holistic understanding of CYP’s presenting needs [6]. High unmet community need and staffing issues in specialist services have led to increasing calls for effective communication between specialists (e.g., psychologists and senior physicians) and frontline staff who interact with CYP in healthcare and in the community (e.g., primary care and educational professionals). This aims to maximize the capacity of a system’s workforce to support the delivery of timely, safe, and effective care and improve outcomes for CYP [4, 7]. Ineffective team communication can result in low quality delivery of care, including delays in care and risks to patient safety [8], particularly for CYP with medical complexity. Digital communication technologies may facilitate the practical implementation of information sharing, consultation, and case management by clinical teams [9, 10], by making team communication more accessible for professionals when attendance at a face-to-face meeting is not possible, and particularly during the COVID-19 pandemic. The relationship between digitally-mediated team communication and performance may be moderated by team and task characteristics [11–14]. A clinical team that problem solves through consultation, bring diverse backgrounds, knowledge, and viewpoints, and where the actions of frontline workers are influenced by the guidance of specialists would be considered to have high team interdependence and working on a task of moderate complexity [11–13]. Based on the propositions made by a conceptual model of communication in digital teams [14], such conditions make effective communication critical for professional practice and improved outcomes, especially for digital clinical teams. For example, the use of digital communication technologies that allow face-to-face contact (e.g., videoconferencing), and that can convey sufficiently rich information and thus enhance communication quality (i.e., media richness theory, [15]), may facilitate performance as well as improve professional satisfaction [14]. It is important to understand the impact of digitally-mediated team communication on professional practice and CYP outcomes.

There is an extensive literature about *face-to-face* multidisciplinary collaboration in CYP services (e.g., [2, 16–20]), with indication of high levels of satisfaction among professionals. There is, however, a paucity of evidence relating to service user experience or clinical outcomes. To date, there is a limited understanding of the outcomes of *digitally-mediated* team communication, especially for responding to CYP’s presenting needs. Systematic reviews of digitally-mediated service models have analysed evidence for practice with CYP [3, 21], and both CYP and adult populations [4, 22–24]. There is relatively less evidence available for CYP compared to adults, and clinical outcomes are typically not assessed in evaluations of service models that focus on digitally-mediated team communication, in contrast to evaluations of service models that involve direct specialist care via digital communication technologies. Digitally-mediated team communication is conceptually different to face-to-face team communication [25], and requires specific measures for process evaluation such as satisfaction with digital communication technologies, especially as there has been some resistance to uptake in health and mental health services (e.g., [26]), with perceived organisational, technical, and security challenges [9, 10]. A focus on CYP service users is important due to specific considerations for practice, such as the type of need (e.g., developmental disorders and early life trauma), the family context, and resources at the CYP site to deliver intervention [3, 17]. In the current review, the focus will be on CYP service users and digitally-mediated team communication.

To the best of our knowledge, this is the first attempt to focus specifically on digitally-mediated team communication in children’s services. Studies reporting on outcomes of digitally-mediated team communication for CYP, although included in the previous reviews [3, 4, 21–24], have not been specifically collated. This data has not been consistently isolated for analysis in studies that evaluate multi-component programmes that combine digitally-mediated team communication with interventions such as education and training or direct specialist care (e.g., [27, 28]). Many studies have been descriptive of the content and process (e.g., [29]) or the characteristics of referred CYP and support recommendations (e.g., [30, 31]), whilst others have not focused predominantly on CYP (e.g., [32, 33]). Nevertheless, it is important to synthesise the relevant outcome data available because digitally-mediated team communication is considered a particularly attractive solution to address issues of cost and capacity in service provision whilst meeting the needs of CYP [3, 32, 34]. With indication that the evidence-base is evolving [3, 22], and the increasing use of digital communication technologies in current practice due to the COVID-19 pandemic, a systematic review of the literature is very timely.

The present systematic review was conducted to explore the outcomes of digitally-mediated team communication for CYP. The research question for this review is: Is there evidence that digitally-mediated team communication facilitates professional practice (as measured post-intervention or compared to baseline or a comparison intervention arm), leads to improved clinical outcomes (compared to baseline or a comparison intervention arm), and demonstrates feasibility and acceptability in children’s health and mental health services?

## Method

We conducted a systematic review following guidelines by the Preferred Reporting Items for Systematic Reviews and Meta-Analyses (PRISMA) [35]. The protocol for this review was registered on Prospero, ID number: CRD42020169733.

### Search Strategy

A detailed systematic search strategy was developed in consultation with a librarian at the University of Bath. Preliminary searches identified a range of terms used in the literature to describe digitally-mediated team communication. We identified four electronic databases (PsycINFO, PubMed, Web of Science, Cochrane Library) to access mental health, psychology-related, and healthcare systems literature. We conducted a systematic search in February 2020, for studies published in English, using a search that contained specific terms (keywords and Medical Subject Headings (MeSH)) relating to team communication (e.g., multidisciplinary communication, interdisciplinary communication, integrated service) and digital communication technologies (e.g., telecommunications, videoconferencing, computer-mediated communication) (i.e., the intervention) and relevant professions (e.g., health personnel, psychologist, general practitioner) (i.e., the users of digital technology). Search terms relating to childhood mental health, developmental, and behavioural conditions (e.g., anxiety, depression, neurodevelopmental disorder) were used for Web of Science as a medical database. We filtered the search results to include the age group 0–18 years (i.e., the recipients of digitally-mediated team communication). The scope was restricted to papers published since 2003, when a current videoconference technology [36] was first released, when the [37] recommended the use of digital technology in services for quality care, and when policies promoting team approaches in the children’s workforce in developed countries were launched, such as the Every Child Matters initiative [38] and the Children Act (2004) in the UK. The search algorithm is outlined in Appendix A. The reference lists of relevant systematic reviews which were identified during screening were checked for additional relevant studies, although none were identified. The reference lists of relevant systematic reviews that were known to the study authors but were not identified during screening were also checked, and two additional relevant studies were identified.

### Eligibility Criteria

To be eligible for inclusion in the systematic review, studies had to meet inclusion/exclusion criteria based on Population, Intervention, Comparison, Outcome, and Study Design (PICO) guidelines [39].

#### Inclusion Criteria

##### Population

Qualified professionals trained in relevant healthcare domains (including psychologists, general practitioners, social workers); CYP (0–18 years of age) service users with any health, mental health, developmental or behavioural condition. We included studies if the majority (> 80%, [40]) of the service user group met the eligibility criteria or if the data was extractable for the sub-sample of the service user group meeting the criteria.

##### Intervention

Programmes or interventions where digital communication technologies (including telephone, email, videoconference technology) were used for communication between two or more professionals for information sharing, consultation, and case management. We included studies of multi-component programmes or interventions if the data was extractable for the digitally-mediated team communication component.

##### Outcomes

Based on the HM Treasury guidance for service evaluation [41], we were interested in exploring the impact and the processes of digitally-mediated team communication. We assessed impact (i.e., the changes that occurred) via professional practice and via outcomes for CYP service users. We assessed processes (i.e., activities involved in an intervention’s implementation) via feasibility and acceptability outcomes. Outcomes were categorised into the three domains in accordance with the telemedicine literature [42]: professional practice outcomes (e.g., professionals’ knowledge, skill, and confidence, technical quality (performance, concordance with best practice guidelines, fidelity to evidence-based protocols, time to reaching a clinical decision; [43]); clinical outcomes (e.g., change in identified clinical symptoms over time); and feasibility and acceptability outcomes (e.g., provider and/or patient satisfaction, the perceived ease (preference, comfort, fit, readiness) of providers to use digital communication technologies, interpersonal quality (team communication)). Studies that included at least one measure of professional practice outcomes and/or at least one measure of clinical outcomes were included in the review. Studies may have assessed feasibility and acceptability outcomes.

#### Exclusion Criteria

##### Study Design

Case studies, literature or systematic reviews, editorials, and conference abstracts were excluded.

### Study Selection

References identified from database searches were de-duplicated and screened in Covidence software [44]. The first author (LJ) screened all titles/abstracts and full-texts, and another member of the research team (EC) independently reviewed 10% of randomly selected titles/abstracts and full-texts. Any disagreement between reviewers at both title/abstract stage and full-text screening was highlighted on the software and resolved through discussion. Inter-rater reliability for title/abstract screening was 100% concordant, and for full-text screening was 100% concordant.

### Quality Assessment

Quality assessment of all selected full-texts was conducted independently by two reviewers (LJ and EC). The Effective Public Healthcare Panacea Project [45] quality assessment guidelines were used for quantitative studies and a quality framework by the UK Cabinet [46] was used for qualitative studies. For mixed-methods studies, we conducted a quality assessment for each study method. Inter-rater reliability for quality appraisal was 88%. Any disagreement between reviewers was resolved through discussion. We rated the overall quality of studies as weak, moderate, or strong. This rating was assessed by the number of weak ratings given for the items within the assessments. Studies with two or more weak ratings were given a weak global rating, studies with one weak rating were given a moderate global rating, and studies with no weak ratings were given a strong global rating. All studies were included regardless of quality due to the paucity of research in this area.

### Data Extraction

The first author extracted the data and discussed with co-authors (MB and AR). A data collection form was used to extract data from the included studies. The form included the following headings: study (authors and year of publication), aims, study design, location, professional participant characteristics, service user participant characteristics, intervention characteristics, outcome measures, and results (professional practice outcomes, clinical outcomes, and feasibility and acceptability outcomes).

### Data Synthesis

Data were synthesised narratively. A narrative data synthesis strategy was selected because the included studies were likely to be heterogeneous in the types of measures, intervention characteristics, and participant characteristics.

## Results

### Search Selection

**Fig. 1:**
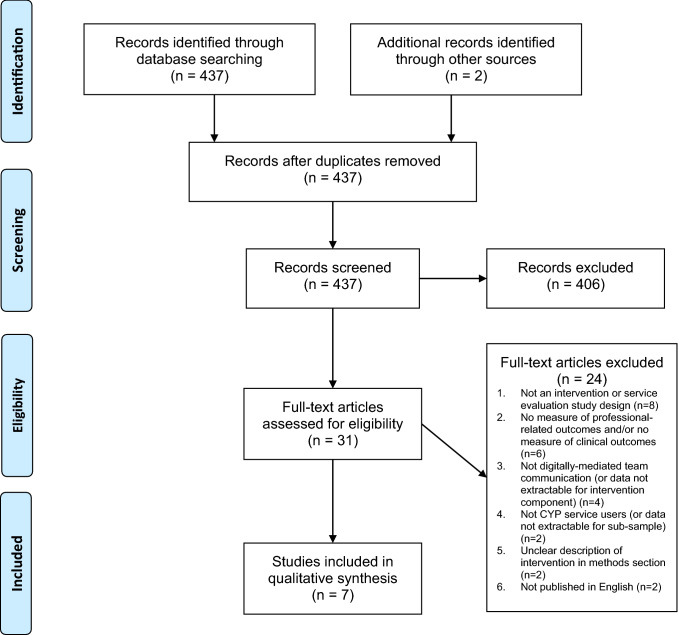
PRISMA flowchart showing selection of studies for systematic review

Our initial electronic database search and identification of additional studies through checking reference lists generated 439 records. After removing two duplicates, 437 records remained for screening. Following title/abstract screening, 31 studies were included for full-text screening, of which seven studies met all inclusion/exclusion criteria. Detailed reasons for exclusion are shown in PRISMA flow diagram (Fig. 1). The most common reason for exclusion at the full-text screening stage was that the paper was ‘not an intervention or service evaluation study design’, with relevant review papers included from title/abstract screening for the checking of reference lists, followed by ‘no measure of professional practice outcomes and/or no measure of clinical outcomes’.

### Result of the Quality Appraisal

Of the seven studies, six underwent quality appraisal using EPHPP [45] ([47–52]) and two underwent quality appraisal using the quality framework for qualitative studies by the UK Cabinet [46] ([50, 53]). Four studies received a weak rating [49–52]. Studies for CYP mental health were low quality due to the use of post-test designs [49, 50, 52], non-validated survey measures [51, 52], lack of description of approach to analysis [50, 51], no discussion of underlying assumptions and ideological perspectives [50], and potential effects of co-interventions on the results [50, 52]. One study received a moderate rating, with a limitation that the outcome assessors were not blinded to the intervention arm [47]. Two studies received a strong rating [48, 53]. Fleischman and colleagues [48] used a controlled clinical trial (CCT) design, relevant confounders were controlled for (including sex, age, body mass index (BMI)), and the follow-up rate was greater than 80%. Volpe and colleagues [53] were given a strong rating for reasons including clear description of the data collection procedure and theoretical framework. The quality assessment is shown in Table 1.

**Table 1:** Quality assessment for studies of digitally-mediated team communication for children and young people

(a) Quality assessment for quantitative methods							
Study	Selection bias	Study design	Confounders	Blinding	Data collection method	Withdrawals and dropouts	Global rating
Butler et al. [47] (2019)	Moderate	Strong	Strong	Weak	Moderate	N/A	Moderate
Fleischman et al. [48] (2016)	Moderate	Strong	Strong	Moderate	Moderate	Strong	Strong
Hilt et al. [49] (2013)	Weak	Weak	N/A	Moderate	Weak	N/A	Weak
Malas et al. [50] (2019)	Weak	Weak	N/A	Moderate	Moderate	N/A	Weak
Straus and Sarvet [51] (2014)	Weak	Moderate	N/A	Moderate	Weak	N/A	Weak
Walter et al. [52] (2019)	Weak	Weak	N/A	Moderate	Weak	N/A	Weak

(b) Quality assessment for qualitative methods										
Study	Findings	Design	Sample	Data collection	Analysis	Reporting	Reflexivity and neutrality	Ethics	Auditability	Global rating
Malas et al. [50] (2019)	Moderate	Strong	Strong	Strong	Weak	Moderate	Weak	Moderate	Moderate	Weak
Volpe et al. [53] (2014)	Strong	Strong	Strong	Strong	Strong	Strong	Strong	Strong	Strong	Strong

(a) Quality assessment conducted using the Effective Public Healthcare Panacea Project [45] guidelines

(b) Quality assessment conducted using a quality framework by the UK Cabinet [46]

### Characteristics of Included Studies

Five studies used digitally-mediated team communication to enhance expertise and practice of frontline staff in community primary care settings [48–52], one study in a community mental health setting [53], and one study in an Emergency Department setting [47]. The seven included studies represent research predominantly located in the United States of America (86%), with one study carried out in Canada [53]. All studies were published in the last decade, of which three were published in the last three years [47, 50, 52]. Two studies were controlled clinical trials (CCTs) [47, 48], and five were service evaluations: three were post-test survey studies [49, 50, 52], one was a pre/post survey study [51], and one was a longitudinal observational study [53]. Of the seven studies, five used quantitative methods [47–49, 51, 52], one used qualitative methods [53], and one used mixed-methods [50]. The respondents were paediatricians or adolescent medicine physicians (n = 6), nurses (n = 5), consultant child psychiatrist (n = 1), social workers (n = 1), child and youth workers (n = 1), community wellness workers (n = 1), and CYP service users and parents in one study [48]. The sample size of professionals in the included studies that report this information, specifically those taking part in the intervention if a CCT, ranged from 20 to 649. Table 2 provides further details of the study characteristics.

**Table 2:** Summary of evidence on digitally-mediated team communication for children and young people

Study	Author, country, study design	Participants	Setting	Service user characteristics	Intervention	Results
Controlled clinical trials						
	Butler et al. [47] (2019), USA, Quantitative	N = 40 physicians, INT = 20, CONT = 20 Physicians: senior resident 50%; junior resident 50%	Emergency Department	Health: computerised infant, paediatric sepsis and paediatric cardiac arrest	*Type of digital technology*: Videoconference *Team characteristics and composition*: Two physicians (senior and junior resident) and two standardized confederate nurses	*Professional practice*: There were no significant differences in scores of overall clinical performance between the INT teams than the CONT teams (*P* = 0.36) There were no significant differences in median time (s) to defibrillation between the INT teams and CONT teams (*P* = 0.55) There were no significant differences between the % of INT teams and % of CONT teams achieving a time of < 180 s to defibrillation (*P* = 0.37) *Clinical*: N/A *Feasibility and acceptability*: There were no significant differences in the teamwork/communication scores between the INT teams and CONT teams (*P* = 0.28) There was significantly higher workload in the INT teams than the CONT teams (*P* = 0.02)
	Fleischman et al. [48] (2016), USA, Quantitative	N = 40 CYP (78%F)^a^ Age M = 14.3 Ethnicity: 88% non-Hispanic white INT 1: N = 19 (74%F) Age M = 14.4 Ethnicity: 95% non-Hispanic white INT 2: N = 21 (81%F) Age M = 14.2 Ethnicity: 81% non-Hispanic white	Community primary care	Health: obesity (BMI ≥ 95th percentile for gender and age)	*Type of digital technology*: Videoconference *Team characteristics and composition*: PCPs (physicians, nurse practitioners, nurses) and obesity specialists (dietitians, psychologist and endocrinologist)	*Professional practice*: N/A *Clinical*: For participants in INT 1, there was no significant change in BMI from baseline to six months (− 0.06, *P* = 0.08) Change in BMI, waist circumference and triceps skinfold did not differ significantly between INT 1 and INT 2 There were no significant changes in blood pressure, physical activity, or diet for INT 1 or INT 2 *Feasibility and acceptability*: Responses on the CYP/parent experience survey^c,^ for participants in INT 1: helpfulness of programme (CYP: *M* = 6.7; *SD* = 2.9; parent: *M* = 7.2; *SD* = 2.9); satisfaction with changes eating and physical activity (CYP: *M* = 7.0; *SD* = 2.5; parent: *M* = 5.5; *SD* = 3.4); satisfaction with weight loss (CYP: *M* = 5.7; *SD* = 3.6; parent: *M* = 5.5; *SD *= 3.6); recommend study to others (CYP: *M* = 8.2; *SD* = 2.1; parent: *M* = 7.9; *SD* = 2.6) There were no significant differences in perceived helpfulness of the programme, satisfaction with changes in eating and physical activity, satisfaction with weight loss, and recommendation of the study to others by CYP and parents between INT 1 and INT 2
Study	Author, country, study design	Participants	Setting	Service user characteristics	Intervention	Results
Service evaluations						
	Hilt et al. [49] (2013), USA, Post-test, quantitative (Survey)	N = 168 PCP responses (out of 970 possible responses (17% response rate) *Profession*: PCPs, including physicians	Community primary care Washington state partnership access line program	Mental health and behaviour: ADHD (52%), anxiety (36%) disruptive behaviour disorder (36%), depression (20%), autism (14%), other including developmental disorder, PTSD, mood disorder bipolar disorder, learning disability, psychotic disorder, sleep disorder	*Type of digital technology*: Telephone *Team characteristics and composition*: 1 PCP and 1 CAP	*Professional practice*: Responses on a Likert scale^d^: “PAL helps me to increase my own skills in the mental health care of my patients” (M = 4.6; SD = 0.7) “PAL helped me to manage my patient’s care” (M = 4.7; SD = 0.6) *Clinical*: N/A *Feasibility and acceptability*: Responses on a Likert scale^d^ showed overall high satisfaction with digitally-mediated team communication (M = 4.6, SD = 0.51) Satisfaction was higher among providers who: reported treating more children in foster care; reported treating more children with psychiatric disorders; and called the program 5 or more times
	Malas et al. [50] (2019), USA, Post-test**,** mixed-method (Survey with Likert scale questions and qualitative items)	N = 649 PCP responses (out of 1475 possible responses (44% response rate) *Profession*: PCPs: paediatricians, obstetrician-gynaecologists, family medicine physicians, nurse practitioners, physician assistants, and certified nurse midwives	Community primary care Michigan collaborative child care (MC3) Program offering several levels of consultation and collaboration including digitally-mediated team communication	Mental health	*Type of digital technology*: Telephone *Team characteristics and composition*: 1 PCP and 1 CAP	*Professional practice*: Confidence in managing their patient’s mental health concern following digitally-mediated team communication = “strongly agree” (M = 1.19; SD = 0.43) Five relevant themes relating to perception and practice changes: (1) Improved comfort and confidence in caring for youth with mental illness (30.9%) (2) Ability to care for youth with complex mental health needs (7.5%) (3) Greater comfort and understanding in the use and monitoring of psychotropics (25.9%) (4) Increased understanding and access to psychotherapy services (2.4%) (5) Improved understanding of non-pharmacologic approaches to management and referral services (3.1%) *Clinical*: N/A *Feasibility and acceptability*: User-friendly nature and efficiency of utilizing the program = “strongly agree” (M = 1.11; SD = 0.33)
						Two relevant themes relating to perceptions: (1) Improved access to mental healthcare for youth (23.1%) (2) Enhanced efficiency of care for youth with mental illness (19.6%) Seven relevant themes relating to critiques or constructive feedback related to digitally-mediated team communication: (1) Lack of comfort and familiarity with telephone consultation process (2.4%) (2) Delays in communication or completing consultation (21.4%) (3) More effective communication modalities to transmit communications (i.e. email, web-based, etc.) (9.5%) (4) Improved follow-up consultation process (clearer process, same CAP providing follow-up, etc.) (4.8%) (5) Conflicting recommendations from different CAP consultations (2.4%) (6) Needing more discretion regarding CAP consultation documentation given sensitive information (2.4%) (7) PCP feeling uncomfortable with increased management of mental health concerns (9.5%)
	Straus and Sarvet [51] (2014), USA, Pre-post test, quantitative (Survey)	*Profession*: PCPs: paediatricians, family physicians, nurse practitioners, physician assistants, behavioural health clinicians, and care coordinators	Community primary care Massachusetts Child Psychiatry Access Project	Mental health and behaviour: ADHD (23%), anxiety (18%), depression (16%), oppositional defiant disorder (6%), autism (4%), other including adjustment disorder, mood disorder, bipolar, PTSD/trauma, OCD, substance use, eating disorder, developmental disability, psychosis, conduct disorder N = 10,553	*Type of digital technology*: Telephone *Team characteristics and composition*: 1 PCP and 1 CAP	*Professional practice*: The percentage of respondents that said they agreed or agreed strongly that they could meet the needs of children with BH problems increased from 8% at baseline to 64% at 5 years *Clinical*: N/A *Feasibility and acceptability*: Satisfaction surveys (1–5 scale) before enrolment and annually indicate that PCPs perceive that access to CAPs has improved, that they are able to receive consultation in a timely manner, and that the consultations are useful
	Walter et al. [52] (2019), USA, Post-test, quantitative (Survey)	N = 66 PCP responses (out of 81 possible responses) PCPs: Paediatrician (84%); Nurse Practitioner (14%); Physician assistant (1%)	Community primary care BH learning community comprises of an educational programme supplemented by digitally-mediated team communication	Mental health and developmental conditions: anxiety (28%), depression (25%), ADHD (16%), behaviour (5%), autism (3%) N = 392 (45%F) CYP aged 0–17 years: N = 317 (81% of patient group)	*Type of digital technology*: Telephone *Team characteristics and composition*: 1 PCP and 1 CAP	*Professional practice*: Respondents agreed that digitally-mediated team communication: Facilitated medication management (93%) Reinforced learning community knowledge (93%). Facilitated decisions about crisis management (85%) Facilitated level of care (84%). Improved the quality of their BH care (91%) *Clinical:* N/A *Feasibility and acceptability*: Respondents agreed that digitally-mediated team communication was convenient (95%) and timely (95%) Respondents agreed that digitally-mediated team communication expedited specialty BH referral (65%)
	Volpe et al. [53] (2014), Canada, Longitudinal, qualitative (Participant observation, Interviews, and Focus Groups)	Health and mental health workers (psychiatric nurses, social workers, child and youth workers, community wellness workers), the consulting psychiatrist, and the lead coordinator	Community mental health TeleLink Mental Health Programme at the Hospital for Sick Children in Toronto comprising of digitally-mediated team communication and education sessions	Mental health and behaviour N = 24^e^	*Type of digital technology*: Videoconference *Team characteristics and composition*: 1 child psychiatrist and varying numbers of frontline workers	*Professional practice*: Two relevant categories: (1) Capacity building (generalisation of case-specific information to other cases, frontline staff offering their own solutions) (2) Overall satisfaction (confidence, supportive, applying knowledge in new ways) *Clinical*: N/A *Feasibility and acceptability*: Two relevant themes: (1) Enhancing the participant experience (comfort levels, uncertainties, understanding of the social, cultural, and systemic context, scheduled time for networking) (2) Ensuring stable and confidential technology (satisfaction, technical/ connection difficulties, confidentiality)

*PCP* primary care provider, *CAP* child and adolescent practitioner, *BH* behavioural health, *INT* intervention, *CONT* control, *M* mean, *SD* standard deviation

^a^Use of 6-month data only before CYP change interventions arms. Intervention arms: (1) digitally-mediated team communication (2) digitally-mediated team communication *plus* direct specialist care via digital communication technologies

^b^Baseline characteristics. 17 participants received intervention arm 1 (digitally-mediated team communication) and 19 participants received intervention arm 2 in the first six months of the trial

^c^10-cm visual analogue scale with 0 indicating “not at all/would not recommend” and 10 indicating “extremely/would highly recommend”

^d^Likert scale with 1 indicating “strongly disagree” and 5 indicating “strongly agree”

^e^Estimated sample size based on approximately two cases discussed per session with 12 sessions in total

### Description of Digitally-Mediated Team Communication Interventions

Three studies used videoconference technology [47, 48, 53] and four studies used telephone [49–52]. The presenting condition of the CYP service user group was anxiety (n = 3), depression (n = 3), attention deficit hyperactivity disorder (ADHD) (n = 3), autism spectrum disorder (n = 3), obesity (n = 1), and paediatric sepsis and paediatric cardiac arrest (computerised) (n = 1). The mental health and behavioural conditions of the service user group were not specified in two studies [50, 53]. The number of service users that the programme or intervention was delivered for in the studies that report this information ranged from 17 to 10,553 CYP. One study was delivered for CYP aged 0–18 as a majority sub-sample of the total service user group [52]. Three studies examined digitally-mediated team communication as one component of a wider programme comprising of education [52, 53] or several levels of consultation and collaboration [50]. Two studies included a comparison group [47, 48].

Butler and colleagues [47] assessed the use of videoconference technology by a team of four professionals, where a senior physician provided consultation in real-time to a junior physician (and two confederate nurses) during a simulated paediatric resuscitation in an emergency department setting. This study compared digitally-mediated team communication to usual care (i.e., face-to-face team communication).

Fleischman and colleagues [48] assessed consultation via videoconference technology regarding CYP obesity in community primary care. This study compared digitally-mediated team communication (intervention 1) to digitally-mediated team communication *plus* direct specialist care via digital communication technology (intervention 2). Outcomes of intervention 1 are the focus of the current review.

Hilt and colleagues [49], Malas and colleagues [50], Straus and Sarvet [51], and Walters and colleagues [52] used telephonic consultation between primary care providers and a child and adolescent practitioner for responding to concerns about mental health in community primary care. The information discussed during consultations included initial assessment and treatment suggestions, and questions about management, services, and referrals to specialist settings for severe clinical cases.

Volpe and colleagues [53] examined the use of videoconferencing technology by a multidisciplinary team, where cases were presented by frontline staff to a consultant child psychiatrist for consultation in a community mental health setting, with varying numbers and composition of frontline staff attending each session.

### Professional Practice Outcomes

Professional practices outcomes include professionals’ knowledge, skill, and confidence, and clinical performance [42, 43].

Five quantitative or mixed-method studies explored professional practice outcomes. Four studies [49–52] found that professionals perceived digitally-mediated team communication to facilitate management of CYP needs and increase knowledge and confidence, with one study reporting that it was perceived to improve quality of care [52]. The pre/post survey study assessed change in the percentage of respondents who agreed that they could meet the needs of children with behavioural health problems, and found that the figure increased from 8% at baseline to 64% at five-year follow-up [51]. One study was a CCT of a simulated paediatric resuscitation and this showed performance outcomes (i.e., overall clinical performance and time to defibrillation) of digitally-mediated team communication that were not significantly different to outcomes of face-to-face team communication [47].

Two studies looked at professional practice outcomes using qualitative methods. Both studies identified themes relating to improved confidence. The mixed-method, post-test survey study used a content analysis of participant responses, and coded nearly one third (30.9%) of responses as related to the theme of improved comfort and confidence in caring for CYP with mental illness [50]. Other common themes related to comfort and understanding of management approaches. The longitudinal study used an interpretivist framework and reported capacity building and overall satisfaction as categories in the analysis, with evidence of generalisation of case-specific information to other cases, frontline staff offering their own solutions, and working in a team supporting confidence in approach [53].

### Clinical Outcomes

One study evaluated clinical outcomes of digitally-mediated team communication for CYP, reporting pre/post changes from baseline to six months [48]. The CCT study did not find significant differences in the primary outcome (BMI at six months). The decrease in BMI and other anthropometry measures did not differ significantly between digitally-mediated team communication (intervention 1) to digitally-mediated team communication *plus* direct specialist care via digital communication technology (intervention 2). There were no significant changes in blood pressure, physical activity, or diet from baseline for both groups at six months.

### Feasibility and Acceptability Outcomes

Feasibility and acceptability outcomes include provider and/or patient satisfaction, the perceived ease of providers to use digital communication technologies, and interpersonal quality (team communication) [42].

Six quantitative or mixed-method studies looked at process outcomes around feasibility and acceptability. Four service evaluations showed high satisfaction among frontline staff, in terms of the convenience, timeliness, and user-friendliness [49–52]. One study reported on CYP service user and parent perceptions at six months [48]. On average, both CYP and parents in the intervention 1 group (digitally-mediated team communication) gave scores of six to seven on a 10-point visual analogue scale for questions about the helpfulness of the programme and satisfaction with changes in health and health behaviours. The study did not find significant differences in perceived helpfulness and satisfaction with changes in health by CYP and parents in the intervention 1 group compared to the intervention 2 group (digitally-mediated team communication *plus* direct specialist care via digital communication technology). One study measured teamwork and workload as process outcomes [47], and found that digitally-mediated team communication was associated with enhanced teamwork but a significantly higher workload compared to face-to-face team communication.

Two studies explored the process of digitally-mediated team communication using qualitative methods [50, 53]. Both studies identified relevant themes relating to technology and confidentiality and participant experience. Themes relating to technology and confidentiality referred to connection difficulties and concerns around documentation of sensitive information. Themes relating to participant experience indicated satisfaction with the approach and enhanced efficiency of care for youth with mental illness; a lack of familiarity but increasing comfort levels with time; uncertainties, inconsistencies, and delays in communication; and a need for contextual understanding by the specialist and scheduled time for team networking. One study suggested some dissatisfaction with increased management of mental health concerns [50].

## Discussion

This is the first systematic review aiming to investigate the evidence for digitally-mediated team communication in children’s health and mental health services. Seven studies were included, six of which evaluated professional practice outcomes and all of which explored feasibility and acceptability outcomes of digitally-mediated team communication. Only one study assessed clinical outcomes and service user experience. Analysis highlighted that while professionals reported some concerns and issues, digitally-mediated team communication was generally valued by professionals for supporting practice and that there was overall satisfaction with the process. There is insufficient evidence to know whether digitally-mediated team communication can bring about improved outcomes in children’s health and mental health services.

The included studies show the perceived value of digitally-mediated team communication for supporting professional practice. Frontline staff working directly with CYP with mental health and behavioural concerns in the community viewed digitally-mediated team communication as improving their delivery of care, knowledge and understanding, and confidence. Having the appropriate level of support and perceiving specialists to be available when working in a challenging environment, as well as drawing on the resources and sense-making of others, might help to explain the development of learning and practice through team communication [54–56]. It is possible that the digital communication technologies used were sufficiently rich in informational value [15, 57], thereby enabling the clinical teams to engage in high quality communication which supported the understanding of the expertise provided by specialists [14]. There was also some observational evidence to suggest that digitally-mediated team communication results in similar, if not improved, clinical performance compared to face-to-face team communication in a controlled setting, although the authors suggest that these results should be interpreted with caution [47]. Overall, preliminary findings indicate that digitally-mediated team communication shows promise as an approach to enhancing the expertise and practice of the workforce.

As with previous reviews of digitally-mediated service models and multidisciplinary collaboration [3, 24, 58]), it is evident that the majority of studies place emphasis on process outcomes of digitally-mediated team communication rather than clinical outcomes. Only one study in the current review measured change in health and service user experience [48]. The results of this study suggest short-term, non-significant clinical improvements. Change in clinical outcomes and perceptions of the help received did not differ significantly between the group receiving digitally-mediated team communication and the group receiving a service model which combined digitally-mediated team communication and direct specialist care via digital communication technology, although the direction of the results suggests that, *without* direct specialist care, digitally-mediated team communication improves outcomes to a lesser extent and there is a lower preference for digitally-mediated team communication by CYP and parents. Service user and family experience can help us to understand the extent of quantitative change in clinical outcomes. However, this study did not measure changes in team communication – in fact, team communication was only assessed by one study in this review [47] – thus it is unclear how communication quality may have contributed to improved clinical outcomes. Of note, it is difficult to determine the causal effects of digitally-mediated team communication and there is a need for long-term follow-up periods for any change in outcomes such as attainment or health and mental health to be realised [16, 17, 20]. Moreover, this suggests that process outcomes should not be overlooked, with inherent importance and for understanding the relationship between digitally-mediated team communication and outcomes for CYP [16].

The quantitative results of this review suggest that digitally-mediated team communication as a process is well-perceived by professionals, whereas the qualitative results present a mixed picture. In general, frontline staff showed high satisfaction with the convenience, timeliness, and user-friendliness of digitally-mediated team communication [49–52]. However, challenges relating to confidentiality concerns and unfamiliar and/or unclear processes were highlighted [50, 53]. Although technical issues were also reported, these were experienced as minor difficulties which contrasts with earlier relevant studies that found significant concerns relating to time delay and picture quality (e.g., [59]). Finally, there was evidence to show a higher workload in terms of mental demand when using digitally-mediated team communication compared to face-to-face team communication [47]. It is possible that high cognitive load did not hinder clinical performance in this study because digitally-mediated team communication was assessed in a single trial, rather than an evaluation of a high number of communication interactions [14].

Given that digitally-mediated team communication is largely perceived as valuable and useful, it is of clinical interest to address the perceived challenges. According to a conceptualisation of digitally-mediated team communication [14], the impact on cognitive load in a digital context, combined with participation experiences that are indicative of difficulties in establishing trust and shared ‘mental models’, may have implications for satisfaction, viability, and performance. In turn, recommendations for team working such as clear governance structures and working processes might be relevant for supporting digitally-mediated team communication [22, 60]. The current review further highlights a pattern of matching the digital communication technology to the team activity and composition. Telephone was used for communication in dyadic teams for consultation [49–52], where simplicity and ease of access might have been prioritised. However, videoconference technology offered a more sophisticated solution for communication in larger and more diverse teams, for the purposes of case management as well as for real-time consultation [47, 48, 53], suggesting that the visual element is important for communication in such clinical teams.

A potentially important consideration for implementation is to match the skills of frontline staff and the use of digitally-mediated team communication to the presenting need of CYP [4, 24], with a respect and recognition of professional roles [34]. Digitally-mediated team communication is often implemented to respond to severe and/or complex needs [30, 33, 50, 61], and while this might be appropriate in settings with highly-skilled frontline staff (e.g., [47]), it might not be sufficient for meeting this level of need in the community [20]. In studies where CYP presented with severe and/or complex needs in the current review, there was some evidence that frontline staff in the community [50], and service users [48], value direct specialist care for CYP and their families. It is possible that staff views not only reflect a desire for CYP to receive the appropriate intensity of support, but may also point to concerns around their workload and professional identity [18, 34], particularly in the context of becoming an extended mental health workforce. With respect to mental health service provision in community settings, digitally-mediated team communication might be more appropriate for managing mild-to-moderate concerns, which is supported by a study with a predominance of moderate cases where nearly two-thirds were considered manageable by frontline staff and the remaining were signposted to specialist support [52].

### Implications for Future Research

The current review points to the following three priorities for future evaluation: (i) outcomes-focussed and longitudinal investigation to gain an understanding of the clinical and cost effectiveness; (ii) process evaluation with assessment of team communication in order to understand causality; and (iii) use of a mixed-method approach, with qualitative investigation to capture rich insight into the detail of the experience of a broader range of stakeholders, including frontline staff, specialists, and service user and family.

The current review identified a gap in the evidence-base relating to digitally-mediated team communication to support frontline staff in non-traditional, naturalistic settings for CYP, such as schools [3, 16, 22, 62]. The predominance of USA and Canadian articles in this review focused on primary care to improve access and service utilisation. The search identified one descriptive study of digitally-mediated team communication to support educational professionals to respond to students with mental health concerns [62], however this article did not meet the criteria for inclusion in the current review. Future research should explore outcomes of digitally-mediated team communication in this area, especially with consideration to the policy interest in Europe and Australia on prevention and early intervention as well as multidisciplinary collaboration.

### Limitations

This review gives a first account of the outcomes of digitally-mediated team communication for supporting CYP. Although a strength of the review was the detailed search criteria to facilitate a synthesis of data relating specifically to digitally-mediated team communication, in a field of diverse and confusing terminology [1, 17], the specificity of the search may have increased the chance of missing relevant research. For example, in the identification of the two additional studies for the current review [49, 51], we became aware that the terms ‘health services accessibility’ and ‘remote consultation’ are used in the USA literature to refer to a coordinated service model that involves rapid access to consultation [63]. In addition to the search limitation, further limitations relating to the methodological quality of the included studies merit consideration. There was a narrow focus on professional practice and process outcomes. This contrasts to broad outcome evaluation in the literature on computerised therapy for service users (e.g., [64, 65]), and relates to the assumption that changes in organisational processes, such as enhanced professional practice facilitated by digitally-mediated team communication, are likely to bring about improved outcomes for CYP and their families as well as economic efficiencies (e.g., [2, 16]). There is currently a lack of high-quality, theory-based research to support this, although it appears that more robust outcome measures and study designs have been used in research for CYP with health or medical conditions compared to mental health and behavioural conditions. Finally, the small number of studies identified and the heterogeneity in the study designs, measures, and the team and service user characteristics limited the synthesis of data.

## Summary

There are increasing calls for digitally-mediated team communication in children’s health and mental health services but it is important to be aware that the evidence base is still in its’ infancy. Systematic review of the literature suggested that digitally-mediated team communication is generally valued by professionals for supporting professional practice and that there is overall satisfaction with the process and service efficiency. Future research should evaluate the clinical and cost outcomes, as well as the process outcomes, of this promising approach in order to understand whether it can effectively meet CYP needs in the longer term and at the same time address current system issues in providing quality care. With the COVID-19 pandemic, digital communication technologies are likely to be increasingly used in children’s services, which makes this a timely systematic review to inform current practice and future service development and evaluation.

## Supplementary Information

Below is the link to the electronic supplementary material.

Supplementary file1 (DOCX 33 kb)

## Data Availability

All data used for the analysis are included in Table 2.
