# Supplementary material for: To What Extent Can Digitally-Mediated Team Communication in Children’s Physical Health and Mental Health Services Bring about Improved Outcomes? A Systematic Review
Source: Child Psychiatry Hum Dev. 2021 May 8;53(5):1018–35. doi: 10.1007/s10578-021-01183-w (PMC8105145; doi:10.1007/s10578-021-01183-w)
Supplement: Supplementary file 1 — Supplementary file1 (DOCX 33 kb) [file 10578_2021_1183_MOESM1_ESM.docx]

**Appendix A: Search Strategy**

**PUBMED**

((("interdisciplinary communication"[MeSH Terms] OR "interdisciplinary communication"[Title/Abstract] OR "inter-disciplinary communication"[Title/Abstract] OR "intersectoral collaboration"[MeSH Terms] OR "intersectoral collaboration"[Title/Abstract]) AND (telecommunications[MeSH Terms] OR telecommunicat*[Title/Abstract] OR tele-communicat*[Title/Abstract] OR videoconferencing[MeSH Terms] OR videoconferenc*[Title/Abstract] OR video-conferenc*[Title/Abstract] OR “Text Messaging”[MeSH Terms] OR “Text Messag*”[Title/Abstract] OR “electronic mail”[MeSH Terms] OR “electronic mail*”[Title/Abstract] OR Computer*[MeSH Terms] OR Computer*[Title/Abstract] OR Telephone[MeSH Terms] OR Telephon*[Title/Abstract] OR “Cell Phone”[MeSH Terms] OR “Cell Phone”[Title/Abstract] OR Smartphone[MeSH Terms] OR Smartphone[Title/Abstract]) AND ("case managers"[MeSH Terms] OR "case manager*"[Title/Abstract] OR "general practitioners"[MeSH Terms] OR "general practitioner*"[Title/Abstract] OR GP[Title/Abstract] OR pediatricians[MeSH Terms] OR pediatrician*[Title/Abstract] OR paediatrician*[Title/Abstract] OR peadiatrician*[Title/Abstract] OR "social workers"[MeSH Terms] OR "social worker*"[Title/Abstract] OR psychologist*[Title/Abstract] OR "patient care team"[MeSH Terms] OR "patient care team*"[Title/Abstract])))**Publication date from 2003/01/01, Child: birth-18 years.**

**PsycInfo**

1 **Keywords**: {Electronic Collaboration} OR {Computer Mediated Communication} OR {Electronic Communication} OR {Virtual Team*} OR {Communication System*} OR {Telecommunications Media} OR {Tele-communications Media} OR {Telephone Systems} OR {Teleconferenc*} OR {Teleconsult*} OR {Videoconferenc*} OR {Tele-conferenc*} OR {Tele-consult*} OR {Video-conferenc*} OR {Computer*} OR {Mobile Phone*} OR {Tablet Computer*} OR {Text Messag*}

2 *AND* **Keywords**: {Professional Personnel} OR {Health Personnel} OR {Mental Health Personnel} OR {Educational Personnel} OR {General Practitioner*} OR {Psychologist*} OR {Clinical Psychologist*} OR {Educational Psychologist*} OR {Clinician*} OR {Counselor*} OR {Psychiatrist*} OR {Psychotherapist*} OR {Speech Therapist*} OR {Social Worker*} OR {Psychiatric Social Worker*}

*AND* **Peer-Reviewed Journals only** *AND* **Year**: 2003 *To* 2020

**Web of Science**

(TS=(“interdisciplinary communication” OR “inter-disciplinary communication” OR “multidisciplinary communication” OR “multi-disciplinary communication” OR “cross-disciplinary communication” OR “intersectoral communication” OR communicat* OR “knowledge transfer” OR “integrated service*”))

OR (TS=(collaborat*) NOT TS=(“collaborative learning”))

AND

(TS=(“Electronic Collaboration” OR E-collaboration OR “Computer Mediated Communication” OR “Computer-Mediated Communication” OR “Electronic Communication” OR “Virtual Team*” OR telecommunicat* OR tele-communicat* OR Teleconferenc* OR tele-conferenc* OR “computer conferenc*” OR videoconferenc* OR video-conferenc* OR Teleconsult* OR tele-consult* OR Computer* OR “Tablet Computer*” OR Telephon* OR “Cell Phone” OR “Mobile Phone” OR Smartphone OR “Text Messag*” OR “electronic mail*” OR email* OR e-mail*))

AND

(TS=(“Professional Personnel” OR “Health Personnel” OR “Mental Health Personnel” OR “Educational Personnel” OR “case manager*” OR “general practitioner*” OR GP OR paediatrician* OR pediatrician* OR peadiatrician* OR “social worker*” OR psychologist* OR “Clinical Psychologist*” OR “Educational Psychologist*” OR Clinician* OR Counselor* OR Psychiatrist* OR Psychotherapist* OR “Speech Therapist*” OR “Psychiatric Social Worker*” OR “patient care team*”))

AND

(TS=(Infan* OR newborn* OR new-born* OR baby OR baby* OR babies OR toddler* OR minors OR minors* OR boy OR boys OR boyhood OR girl* OR kid OR kids OR child OR child* OR children* OR schoolchild* OR “school child*” OR adolescen* OR juvenil* OR youth* OR teen* OR pubescen* OR pediatrics OR pediatric* OR paediatric* OR peadiatric* OR prematur* OR preterm*))

AND

(TS=(“Mental health” OR “Mental Health Service*” OR “Mental Disorder*” OR “Psychological Disorder*” OR “Affective Disorder*” OR “Anxiety Disorder*” OR Anxi* OR “Generalised Anxiety Disorder*” OR “Panic Disorder*” OR Phobia* OR “Separation Anxiety Disorder*” OR “Social Anxiety*” OR Depress* OR “Major Depression Disorder*” OR “Behaviour Disorder*” OR “Development Disorder*” OR “Neurodevelopmental Disorder*” OR “Autism Spectrum Disorder*” OR “Attention Deficit Disorder*” OR “Learning Disorder*” OR “Eating Disorder*” OR “Speech Disorder*” OR “Speech and Language Disorder*” OR “Adjustment Disorder*” OR Trauma* OR “Attachment Disorder*” OR “Posttraumatic Stress Disorder*” OR “Post-traumatic Stress Disorder*” OR “Personality Disorder*”))

**Cochrane Library**

#1 MeSH descriptor: [Interdisciplinary Communication] this term only

#2 MeSH descriptor: [Intersectoral Collaboration] this term only

#3 MeSH descriptor: [Delivery of Health Care, Integrated] this term only

#4(("interdisciplinary communication") or ("intersectoral collaboration") or ("inter-disciplinary communication") or ("multidisciplinary communication") or ("multi-disciplinary communication") or ("cross-disciplinary communication") or ("knowledge transfer") or ("integrated health care")):ti,ab,kw

#5 #1 or #2 or #3 or #4

#6 MeSH descriptor: [Telecommunications] this term only

#7 MeSH descriptor: [Electronic Mail] this term only

#8 MeSH descriptor: [Telephone] this term only

#9 MeSH descriptor: [Smartphone] this term only

#10 MeSH descriptor: [Cell Phone] this term only

#11 MeSH descriptor: [Text Messaging] this term only

#12 MeSH descriptor: [Videoconferencing] this term only

#13 MeSH descriptor: [Computers] this term only

#14 MeSH descriptor: [Computers, Handheld] this term only

#15 ((“Electronic Collaboration”) OR (E-collaboration) OR (“Computer Mediated Communication”) OR (“Computer-Mediated Communication”) OR (“Electronic Communication”) OR (“Virtual Team*”) OR (telecommunicat*) OR (tele-communicat*) OR ("electronic mail*") OR (email*) OR (e-mail*) OR (telephon*) OR ("cell phone*") OR ("mobile phone") OR ("text messag*") OR (videoconferenc*) OR (video-conferenc*) OR (Teleconferenc*) OR (tele-conferenc*) OR (teleconsult*) OR (“computer conferenc*”) OR (computer*) OR ("tablet computer*") OR (smartphone*)):ti,ab,kw

#16 #6 #7 or #8 or #9 or #10 or #11 or #12 or #13 or #14 or #15

#17 MeSH descriptor: [Patient Care Team] this term only

#18 MeSH descriptor: [Counselors] this term only

#19 MeSH descriptor: [Health Personnel] this term only

#20 MeSH descriptor: [Educational Personnel] this term only

#21 ((“Professional Personnel”) OR (“Health Personnel”) OR (“Mental Health Personnel”) OR (“Educational Personnel”) OR (“case manager*”) OR (“general practitioner*”) OR (GP) OR (paediatrician*) OR (pediatrician*) OR (peadiatrician*) OR (“social worker*”) OR (psychologist*) OR (“Clinical Psychologist*”) OR (“Educational Psychologist*”) OR (Clinician*) OR (Counselor*) OR (Psychiatrist*) OR (Psychotherapist*) OR (“Speech Therapist*”) OR (“Psychiatric Social Worker*”) OR (“patient care team*”)):ti,ab,kw

#22 - #17 or #18 or #19 or #20 or #21

#23 MeSH descriptor: [Adolescent] this term only

#24 MeSH descriptor: [Child] this term only

#25 MeSH descriptor: [Infant] this term only

#26 MeSH descriptor: [Child, Preschool] this term only

#27 ((Infan*) OR (newborn*) OR (new-born*) OR (baby) OR (baby*) OR (babies) OR (toddler*) OR (minors) OR (minors*) OR (boy) OR (boys) OR (boyhood) OR (girl*) OR (kid) OR (kids) OR (child) OR (child*) OR (children*) OR (schoolchild*) OR (“school child*”) OR (adolescen*) OR (juvenil*) OR (youth*) OR (teen*) OR (pubescen*) OR (pediatrics) OR (pediatric*) OR (paediatric*) OR (peadiatric*) OR (prematur*) OR (preterm*)):ti,ab,kw

#28 - #23 or #24 or #25 or #26 or #27

#29 - #5 and #16 and #22 and #28
